# Development of a multiplicative, multi-attribute utility function and eight single-attribute utility functions for the Health Utilities Index Mark 3 in Japan

**DOI:** 10.1186/s41687-020-00188-8

**Published:** 2020-04-03

**Authors:** Shinichi Noto, Takeru Shiroiwa, Makoto Kobayashi, Tatsunori Murata, Shunya Ikeda, Takashi Fukuda

**Affiliations:** 1 Department of Rehabilitation, Niigata University of Health and Welfare, 1398 Shimami, Kita-ku, Niigata City, Niigata Japan; 2 Center for Outcomes Research and Economic Evaluation for Health (C2H), National Institute of Public Health, Wako, Saitama Japan; 3 CRECON Medical Assessment Inc., The Pharmaceutical Society of Japan, Nagai Memorial, 12-15 Shibuya 2-chome, Shibuya-ku, Tokyo, 150-0002 Japan; 4 Department of Public Health, School of Medicine, International University of Health and Welfare, 4-3 Kozunomori, Narita, Chiba 286-8686 Japan

**Keywords:** Health utilities index, Health utilities index mark 3, Preference-based measure, Standard gamble, Health-related quality of life, Quality-adjusted life year

## Abstract

**Background:**

The Health Utilities Index Mark 3 (HUI3) is a generic multi-attribute, preference-based system for assessing health-related quality of life. It is widely used overseas as an outcome measure and for estimating quality-adjusted life years. We aimed to estimate a multi-attribute and eight single-attribute utility functions for the HUI3 system based on community preferences in Japan. We conducted two preference surveys in this study. The first survey was designed to estimate a model of utility function and collect preference scores, and the second survey was designed to evaluate predictive validity of the utility function and provide independent scores. Values obtained from the feeling thermometer and standard gamble scores obtained from using a chance board were included in the preference scale. We recruited 1043 respondents (age: 20–79 years) from five cities in Japan through the general population classified by sex and age groups. Respondents were further randomly divided into a modeling group (*n* = 774) and a direct group (*n* = 263).

**Results:**

We acquired the estimation for eight single-attribute and a global multi-attribute utility function. The minimum expected multi-attribute utility score was − 0.002. The intraclass correlation coefficient between the directly measured utility score and the score generated by multi-attribute function in terms of 53 health conditions was 0.742.

**Conclusions:**

The HUI3 scoring function developed in Japan has a strong theoretical and empirical basis. It will be useful in future to predict the directly measured score of health technology assessments in Japan.

## Background

In Japan, a new decision-making process for determining the prices of medical technology has started from fiscal year 2019 [1]. The first officially approved guideline for the economic evaluation of drugs and medical devices has been developed for the analytical process in Japan [2]. The guideline describes that the preference-based measure (PBM) with scoring algorithm developed in Japan should be used when assessing a new quality of life for economic evaluation. However, only few PBMs with scoring algorithm have been developed in Japan (e.g., the 3-level version of the EQ-5D and 5-level version of the EQ-5D [EQ-5D-5 L]). According to the guideline in many other countries (the United Kingdom [3], Canada [4], France [5], Netherlands [6], and Australia [7]), the usage of EQ-5D is mainly recommended as a PBM. However, the guideline in some other countries (Canada [4], France [5], and Australia [7]) recommends the use of the Health Utilities Index Mark 3 (HUI3) for estimating quality-adjusted life years.

The HUI3 is a generic multi-attribute, preference-based system for assessing health-related quality of life (HRQL) [8] and comprises two complementary components [8, 9]: the first is a multi-attribute health-status classification system used to describe health status and the second is a multi-attribute utility function used to value the health status assessed through the multi-attribute health-status classification system of the previous component. The system defines 972,000 unique health statuses, as it focuses on eight attributes (vision, hearing, speech, ambulation, dexterity, emotion, cognition, and pain or discomfort), with each stratified into five to six functional levels. A single-attribute scoring function generates scores for each attribute in the range of 0.00 (the most impaired) to 1.00 (no impairment). The original HUI3 multi-attribute function from Canada generates scores in the range of − 0.36 (most impaired, the all-worst HUI3 health state) to 0.00 (being dead) or to 1.00 (perfect health) [9]. The HUI3 has been utilized to evaluate health conditions and HRQL of several patient groups with chronic diseases, and it is a reliable and relevant scale [10–15].

The health-status classification system represents an individual’s health at a certain point based on eight attributes in health. There are five to six levels per attribute, ranging from a normal disability level to a severe disability level. For instance, there would be various ranges for pain from “free of pain and discomfort” to “severe pain that prevents most activities.” The scoring function based on a directly measured preference score (community preference) obtained from the random samples of respondents in a general population survey provides the utility score for all defined health states by the health-status classification system, from the conventional dead to perfect health scale. The HUI3 scoring function system is based on the multi-attribute utility theory (MAUT) [16, 17]. In the HUI3, the preference score is measured using standard gambling (SG) as the gold standard from MAUT. Precisely, visual analog scale (VAS)-measured values are converted to SG-measured preferences. The HUI3 health-status classification and preference scoring systems have been verified in various ways by researchers worldwide [10–13]. Direct evidence for the international generalizability of the HUI3 utility scoring function has been reported by LeGalès et al. [18], in that the HUI3 multi-attribute function from France is very similar to the original one from Canada.

This study aimed to develop one multi-attribute and eight single-attribute utility functions for the HUI3 system based on community preferences in Japan in order to use the Japanese economic evaluation in accordance with the unique method.

## Methods

### Study design and data collection

The design of the HUI3 preference measurement study included two complementary surveys: a survey to collect measurements required for fitting HUI3 multi-attribute utility functions—the HUI3 Modeling survey (HUI3-M)—and an associated survey to collect direct utility measures for 53 states, including states prevalent in the general population (HUI3-D). The HUI3-D provides a valuable commensurate data set for assessing inter-survey or external agreement of HUI3 utility scores. These surveys were conducted in a face-to-face interview.

Both surveys were conducted in five cities in Japan (Sapporo, Tokyo, Nagoya, Osaka, and Fukuoka). These cities are representative of various regions in Japan and are geographically dispersed. All respondents from 20 to 79 years of age were recruited based on snowball sampling by a research company (ANTERIO Inc.). The HUI3-M preference survey collected value and utility measurements from 774 respondents. Sets of health states were randomly allocated to respondents according to strata. Health state strata were defined as follows: scale anchor states (pits [V6, H6, S5, A6, D6, E5, C6, and P5], dead, and perfect health), methodological marker states (MA [V2, H1, S1, A1, D1, E1, C1, and P3], MB [V2, H1, S1, A3, D1, E2, C1, and P3], and MC [V2, H1, S1, A1, D1, E2, C3, and P5], single-attribute states, and block states. The HUI3-D preference survey collected value and utility measurements from 263 respondents. As in the HUI3-M survey, sets of health states were randomly allocated to HUI3-D respondents according to strata. Health state strata for the HUI3-D survey were defined as follows: scale anchor states, methodological marker states, most prevalent states, and less prevalent states. For the HUI3-M survey, the number of respondents providing value and utility measures varied by health state strata; therefore, precision of the mean preference scores varied by strata.

Value scores were measured using the two-sided feeling thermometer developed by Furlong et al. [19], a prop for eliciting preference scores based on the VAS technique. Standard gamble questions were administered using a modified version of the original chance board prop as follows: in the first step, the interviewee was with certainty in the described health state; in the second step, they were in the best possible state with a certain probability or in the state they considered to be the worst possible one with complementary probability. Different probability values were proposed in an iterative manner until the interviewees stated that they felt indifferently toward both propositions. This last set of data enabled us to establish the function for transforming the values into utilities. Interviews were conducted by 50 interviewers whom we trained in the specific field of preference elicitation in each region. Interviewers used specifications that included both instructions for managing the interviews and those to be read aloud to the interviewees.

### Statistical analysis

Direct preference measures, both values and utilities, are summarized using various statistics: the 10% trimmed mean (5% trimmed off each end of the distribution), standard deviation, minimum, and maximum. The trimmed mean was selected, rather than the median or mode, to maintain most statistical properties associated with using mean-type estimates while reducing the effects of outlier scores on the estimates of central tendency for distributions of the health state preference score with skewed distributions. The person-mean score was defined as the trimmed mean for a specific health state.

The underlying theory of the multiplicative, multi-attribute utility function was described previously by Keeny and Raiffa [20]. The general form for an eight-attribute multiplicative function is as follows:

1 $$ 1+c=\prod \limits_{j=1}^8\left(1+c\ast cj\right) $$

The subscripts (j) indicates a sub-group of attributes.

where $\prod \limits_{j=1}^8$ is the product of all (1 + c*c_*j*_) from *c*_*1*_ to *c*_*8*_.

Respondents were classified into two groups according to the state that each respondent selected as the lowest anchor state when using the feeling thermometer (group A respondents reported pits to be equally or less preferable to dead, and group B respondents reported dead less preferable than pits). Person-mean value scores were calculated for groups A (person-mean (A)) and B (person-mean (B)) scores. Overall person-mean disutility scores were used to fit a multi-attribute disutility function (MADUF), with the scale defined such that perfect health = 0.00 and pits = 1.00 (2), and the MADUF was converted into a multi-attribute utility function (MAUF), with the scale defined such that pits = 0.00 and perfect health = 1.00 (3). Then, the MAUF on the pits/PH scale was converted to a MAUF on the conventional dead = 0.00 to PH = 1.00 scale (4).

#### Formula (pits/PH scale)

MADUF:

2 $$ \overline{u}=\Big[1/c\left\lceil \prod \limits_{j=1}^8\left(1+c\ast cj\ast {\overline{u}}_j\right)-1\right\rceil $$

MAUF:

3 $$ u=1-\overline{u} $$

#### Conversion to the dead/PH scale

4 $$ {\displaystyle \begin{array}{l}{\overline{u}}^{\ast }=\overline{u}/{\overline{u}}_{Dead}\\ {}{u}^{\ast }=1-{\overline{u}}^{\ast}\end{array}} $$

Each respondent’s value scores for the single-attribute (including corner) states were normalized such that the least desirable health state was assigned a value score of 0.00 and the most desirable health state was assigned a value score of 1.00. Next, respondent preference measures (i.e., value and utility scores) were classified into one of two groups: person-mean (A) or person-mean (B). The person-mean single-attribute disutility scores provide the ${\overline{u}}_j$ ‘s. The *c*_*j*_ ’s are the disutility scores for each of the lowest attribute-level states (i.e., the corner states) on the pits/PH scale, and c was calculated by iteratively solving the equation.

Finally, external agreement (i.e., the extent to which each model can predict utility scores for a group respondents other than the group whose preference scores were used to develop the model) was assessed by comparing the utility scores calculated using the MAUF for each of the 53 health states (marker and 50 other states) to the mean of directly measured utility scores for these states, as reported by respondents in the HUI-D preference survey. Agreement between utility scores by SG and scores was assessed using a two-way mixed model intraclass correlation (ICC) in which the SG and HUI3 MAUF scores were treated as fixed effects and interactions between the participant and instrument were treated as random effects [21]. The ICC estimates the proportion of between-subject variation in relation to total variation, where 1 represents perfect agreement and 0 indicates no agreement at all. A coefficient < 0.40 was considered as poor agreement [22]. Statistical analyses were performed using SAS 9.4.

## Results

**Fig. 1:**
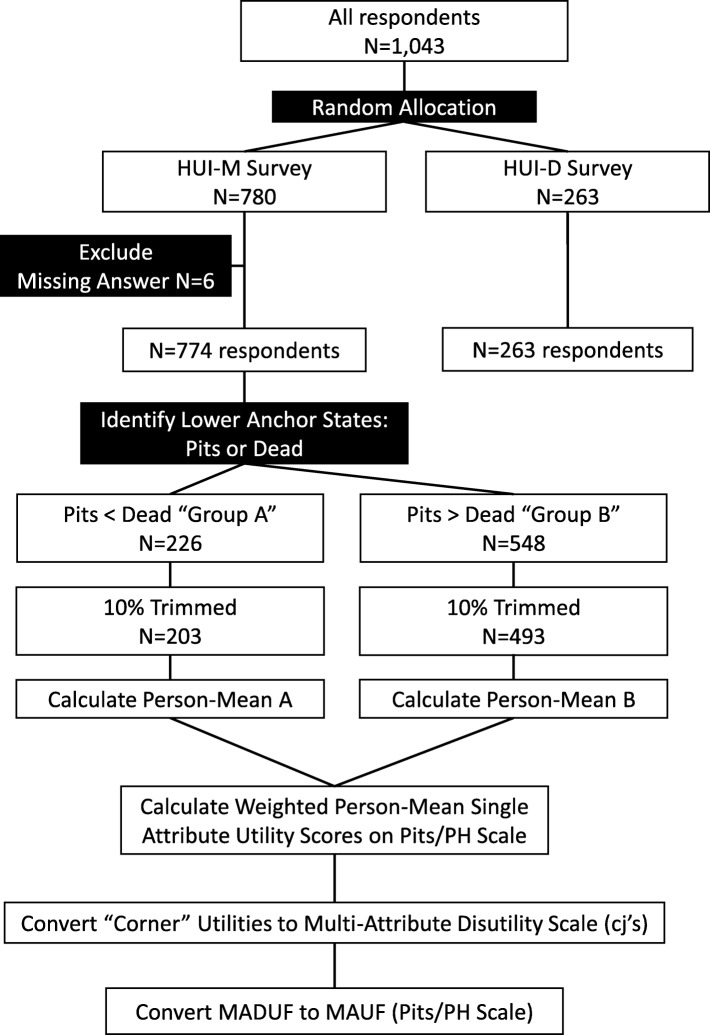
Flowchart of the survey

Flowchart of the survey is shown in Fig. 1. From February to March 2019, 1043 respondents were interviewed by 50 interviewers. Of these, we could not obtain scores because of missing answers on VAS (*n* = 6). As a result, the analysis set included data from 1037 respondents: 774 in the HUI-M and 263 in the HUI-D. Table 1 presents basic characteristics of the respondents (sex, age, location, type of employment, marital status, educational background, and annual household income). We sampled the same number of respondents from each age category; therefore, the groups had almost the same sex ratio. Married and unmarried people accounted for 65.2% and 24.7% of the population in the HUI-M and 65.0% and 24.0% of the population in the HUI-D, respectively. The background of the respondents was comparable with that of the general population.

**Table 1:** Basic socio-demographic characteristics of the respondents

	HUI-M Population (*N* = 774)		HUI-D Population (*N* = 263)	
Gender				
Male	390	50.4%	129	49.0%
Female	384	49.6%	134	51.0%
Age				
20 < = < =29	127	16.4%	44	16.7%
30 < = < =39	128	16.5%	46	17.5%
40 < = < =49	127	16.4%	43	16.3%
50 < = < =59	130	16.8%	45	17.1%
60 < = < =69	130	16.8%	42	16.0%
70 < = < =79	132	17.1%	43	16.3%
Location				
Sapporo	156	20.2%	51	19.4%
Tokyo	154	19.9%	54	20.5%
Nagoya	153	19.8%	54	20.5%
Osaka	155	20.0%	53	20.2%
Fukuoka	156	20.2%	51	19.4%
Employment				
Employed or self-employed	468	60.5%	179	68.1%
Retired	50	6.5%	18	6.8%
Student	41	5.3%	6	2.3%
Homemaker	155	20.3%	41	15.6%
Leave	13	1.7%	4	1.5%
Others	47	6.1%	15	5.7%
Marital status				
Married	505	65.2%	163	62.0%
Unmarried	191	24.7%	63	24.0%
Divorced	47	6.1%	24	9.1%
Bereaved	29	3.7%	13	4.9%
Others	2	0.3%	0	0.0%
Education				
Junior Highschool	19	2.5%	10	3.8%
Highschool	292	37.7%	88	33.5%
College etc.	205	26.5%	74	28.1%
University	248	32.0%	88	33.5%
Graduate school	10	1.3%	3	1.1%
Household income				
<JPY 2mil	59	7.6%	27	10.3%
JPY 2 mil<= < 4 mil	170	22.0%	60	22.8%
JPY 4 mil<= < 6 mil	216	27.9%	61	23.2%
JPY 6 mil<= < 10 mil	194	25.1%	63	24.0%
JPY 10 mil<= < 15 mil	62	8.0%	26	9.9%
JPY 15 mil <=	15	1.9%	5	1.9%
Refused, Unknown	58	7.5%	21	8.0%

The person-mean (A) value/utility model was fitted using the person-mean (A) value and person-mean (A) utility scores for states MA, MB, and MC. Each person-mean score is reported in Table 2. The person-mean (A) value and utility scores for these states were each based on 226 observations. Scores for the MA, MB, and MC states were 0.725 0.659, and 0.487, respectively. The person-mean (A) fitted value/utility relationship was as follows:

$$ \mathrm{u}={\mathrm{v}}^{0.7467} $$

**Table 2:** Scores for three marker states in two groups

	10% trim mean (VAS)		10% trim mean (SG)	
	Person-mean (A) (*N* = 226)	Person-mean (B) (*N* = 548)	Person-mean (A) (*N* = 208)	Person-mean (B) (*N* = 489)
MA^a^	83.8	85.3	0.725	0.688
MB	62.4	64.1	0.659	0.610
MC	35.6	35.9	0.487	0.455
Dead	16.2	–	0.315	–
Pits	–	4.3	–	0.113

^a^MA [V2, H1, S1, A1, D1, E1, C1, and P3], MB [V2, H1, S1, A3, D1, E2, C1, and P3], MC [V2, H1, S1, A1, D1, E2, C3, and P5] and Pits [V6, H6, S5, A6, D6, E5, C6, and P5]

The fitting process used straight-line regression through the origin on the natural log transformations of person-mean (A) value and utility scores. The fit yielded an R^2^ of 0.8687, not corrected for the mean.

The person-mean (B) value/utility model was also fitted using person-mean (B) value and person-mean (B) utility scores for states MA, MB, and MC. Person-mean (B) value and utility scores for these states were each based on 548 observations. Scores for the MA, MB, and MC states were 0.688, 0.610, and 0.455, respectively. The person-mean (B) fitted value/utility relationship was as follows:

$$ \mathrm{u}={\mathrm{v}}^{0.8437} $$

The R^2^ was 0.8437, not corrected for the mean.

Table 3 presents the single-attribute utility functions on the pits = 0.00/PH = 1.00 scale and single-attribute utility functions on the lowest level = 0.00/highest level = 1.00 scale.

**Table 3:** Single-attribute utility function for Japanese HUI3

Level	Vision	Hearing	Speech	Ambulation	Dexterity	Emotion	Cognition	Pain
1	1.00	1.00	1.00	1.00	1.00	1.00	1.00	1.00
2	0.96	0.89	0.87	0.89	0.90	0.91	0.89	0.92
3	0.77	0.75	0.56	0.73	0.71	0.63	0.83	0.77
4	0.58	0.53	0.33	0.44	0.42	0.28	0.58	0.35
5	0.31	0.35	0.00	0.24	0.17	0.00	0.29	0.00
6	0.00	0.00	–	0.00	0.00	–	0.00	–

For measurement and computational convenience, the multiplicative, multi-attribute utility function (Eq. 1) was fitted using disutility scores [19] (disutility = 1 − utility). The disutility function was fitted on the perfect health = 0.00 to most disabled = 1.00 scale. The estimates on this scale were as follows: c1 = 0.58, c2 = 0.46, c3 = 0.54, c4 = 0.58, c5 = 0.57, c6 = 0.61, c7 = 0.63, c8 = 0.53, c = − 0.998, and Σci = 4.50 (the disutility parameters, ci, correspond to the utility parameters, kj, in Eq. (1); c corresponds to k). It is noteworthy that the sum of the ci was 4.50, and the value for c was − 0.998. A simplified format of the scoring function, converted from disutility to utility and transformed using the dead/perfect health scale, appears in Table 4. MAUF was as follows:

$$ \mathrm{u}=1.003\times \left(\mathrm{b}1\times \mathrm{b}2\times \mathrm{b}3\times \mathrm{b}4\times \mathrm{b}5\times \mathrm{b}6\times \mathrm{b}7\times \mathrm{b}8\right)-0.003. $$

**Table 4:** Multi-attribute utility function for Japanese HUI3

Level	Vision	Hearing	Speech	Ambulation	Dexterity	Emotion	Cognition	Pain
1	1.00	1.00	1.00	1.00	1.00	1.00	1.00	1.00
2	0.97	0.95	0.93	0.94	0.94	0.94	0.93	0.96
3	0.87	0.88	0.76	0.84	0.84	0.77	0.90	0.88
4	0.76	0.78	0.64	0.67	0.67	0.56	0.74	0.66
5	0.60	0.70	0.46	0.55	0.53	0.39	0.55	0.47
6	0.42	0.54	–	0.42	0.43	–	0.37	–

u = 1.003*(b1*b2*b3*b4*b5*b6*b7*b8)-0.003

Pits score = −0.002

Scores estimated using the multi-attribute utility function were defined on a scale such that the minimum score was − 0.002 (most disabled), dead was 0.00, and the maximum (perfect health) score was 1.00. This approach to model fitting did not involve analysis of variance, and this distinction was an important design factor in selecting the appropriate methods for assessing the performance of the MAUF.

**Fig. 2:**
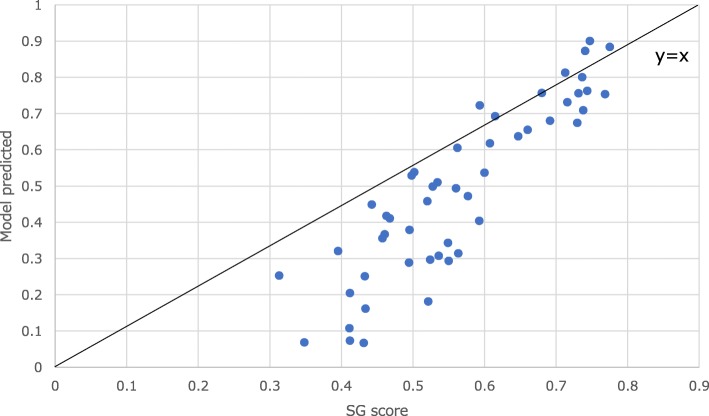
External agreement (SG scores vs scores using MAUF)

A high level of intra-survey (HUI-M) agreement was observed between the person-mean scores and scores used in the MAUF model (ICC = 0.756). The inter-survey agreement statics are presented in Fig. 2 for 53 health states reported to be prevalent in the general population. The ICC point between directly measured utility scores and scores generated by the multi-attribute function estimate was 0.742 (95%CI = 0.592–0.843).

## Discussion

In this study, the preference measurement survey included two widely used preference elicitation instruments: a VAS known as the feeling thermometer and the standard gamble implemented using the chance board. Feeny et al. [23] reported that the use of props and face-to-face interviews by well-trained interviewers have important effects on eliciting the preference score. The use of these well-tested props also had a mutually beneficial effect on the face-to-face interview survey in our study.

The multiplicative, multi-attribute HUI3 scoring function is based on a well-established theory and on the gold standard method for measuring preferences—the standard gamble. The multiplicative functional form accommodates important interactions in the preferences among attributes. Our result was almost consistent with that obtained in Canada for the HUI3 [9]. The similarities of our protocol with those chosen to construct the HUI3 multi-attribute utility function in Canada enabled us to make an international comparison of our results.

Scores estimated using the multi-attribute utility function are defined on a scale such that the minimum score is − 0.002, similar to that of the EQ-5D-5 L pits state [55555], which was − 0.035 for Japan [24]. Despite the existence of these similarities between the pits score by the Japanese HUI3 and that by the Japanese EQ-5D-5 L, the difference between the pits score by the Japanese HUI3 (minimum score = − 0.002) and one by the original Canadian function (minimum score = − 0.36) is very large probably due to the preference for worse than dead. However, at this time, it is difficult to make a definitive conclusion regarding this issue, so further research is needed.

The HUI3 has more attributes than the EQ-5D-5 L, which has five attributes (domains) [25]. The weakness of the EQ-5D-5 L, as reported by Brazier et al. [26], is a lack of domains for vision and hearing and cognition and dementia. The HUI3 has the potential to compensate for the weakness of the EQ-5D-5 L, and the development of the function in Japan using our work is sensible.

A high level of agreement was observed between scores for 53 health states generated by the function and directly measured scores for the same states from a direct survey. The multi-attribute function, which was developed in Japan, has strong theoretical and empirical foundations and performs well in predicting directly measured scores for health technology assessment in Japan.

To the best of our knowledge, the work presented herein is the first attempt at revealing individual preferences for health states using VAS and SG methods performed in a general Japanese population.

## Conclusions

We developed the first multiplicative, multi-attribute utility function-based standard gamble in Japan. The Japanese HUI3 MAUF seems to perform very well. The level of intra- and inter-survey agreement scores might be interpreted as evidence of both validity and reliability. Our work will be useful in establishing cost-effectiveness analyses in Japan where new decision-making processes of the pricing of health technologies has started.

## Data Availability

Data sharing is not applicable to this article as no datasets were generated or analyzed during the current study.

## Abbreviations

HUI3: Health Utilities Index Mark 3
PBM: Preference-based measure
EQ-5D-5 L: 5-level version of the EQ-5D
HRQL: Health-related quality of life
MAUT: Multi-attribute utility theory
SG: Standard gambling
VAS: Visual analog scale
HUI3-M: HUI3 modeling survey
HUI3-D: HUI3 direct survey
MADUF: Multi-attribute disutility function
MAUF: Multi-attribute utility function
ICC: Intraclass correlation
